# Supplementary material for: Dissecting the roles of Haspin and VRK1 in histone H3 phosphorylation during mitosis
Source: Sci Rep. 2022 Jul 1;12:11210. doi: 10.1038/s41598-022-15339-8 (PMC9249732; doi:10.1038/s41598-022-15339-8)
Supplement: Supplementary file 4 — Supplementary Information. [file 41598_2022_15339_MOESM4_ESM.pdf]

Supplementary Information

Dissecting the roles of Haspin and VRK1 in Histone H3 phosphorylation during mitosis

Tyrell N. Cartwright, Rebecca J. Harris, Stephanie K. Meyer, Aye M. Mon, Nikolaus A. Watson, Cheryl Tan, Agathe Marcelot, Fangwei Wang, Sophie Zinn-Justin, Paula Traktman, Jonathan M.G. Higgins

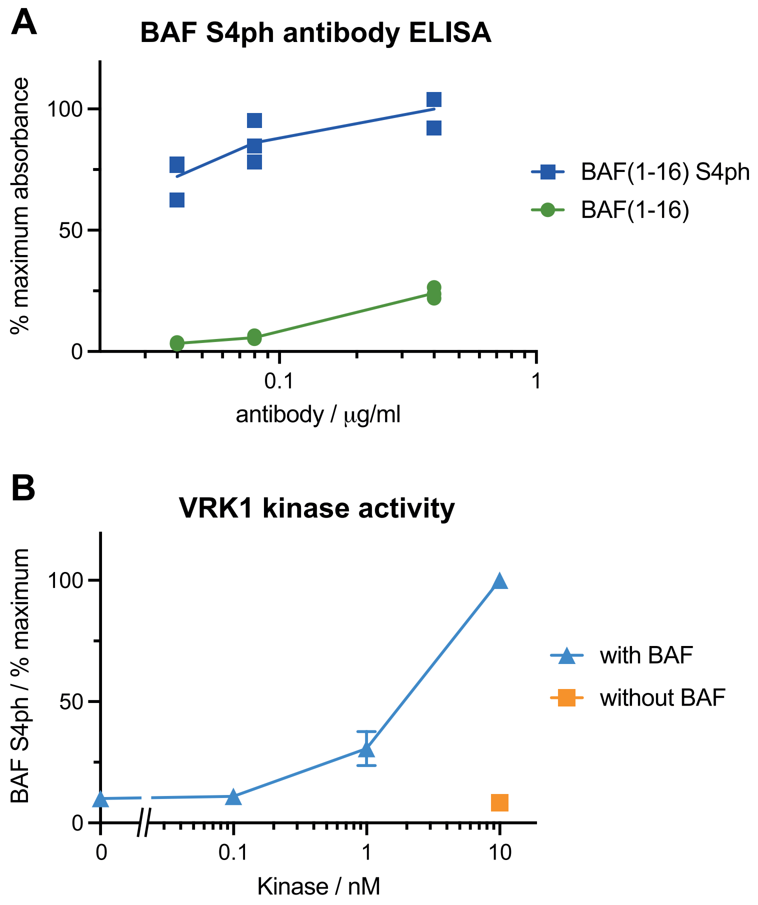

Supplementary Figure 1
Recombinant VRK1 phosphorylates recombinant human BAF.
A. Confirmation of BAF S4ph antibody selectivity by ELISA on BAF(1-16) peptides with and without phosphorylation at Ser-4. The experiment was done once and the values of 3 technical replicates are shown.
B. Recombinant VRK1 phosphorylates recombinant BAF (in which all 4 cysteines are mutated to alanine) at Ser-4, as detected by BAF S4ph antibodies. Error bars show means +/- SD (n =3 independent experiments).

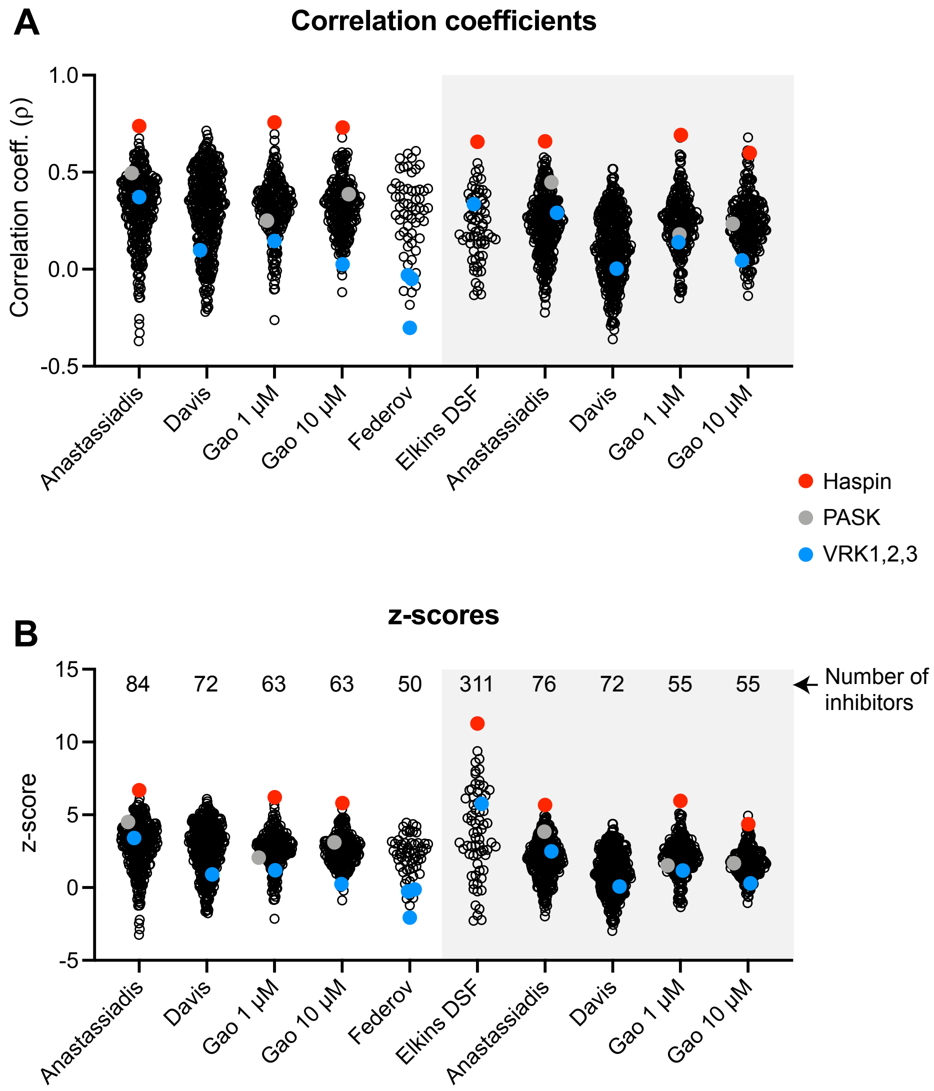

Supplementary Figure 2
Results of KiPIK screens for mitotic H3T3 kinases in HeLa cell extracts.
A. Correlation coefficients for all kinases tested. **B**. z-scores for all kinases tested.
The screens shown on a gray background were previously published[65]. Z-scores allow a more direct comparison of the robustness of kinase ranking between screens which accounts for the number of inhibitors used (shown at the top of the plot), as described[65].

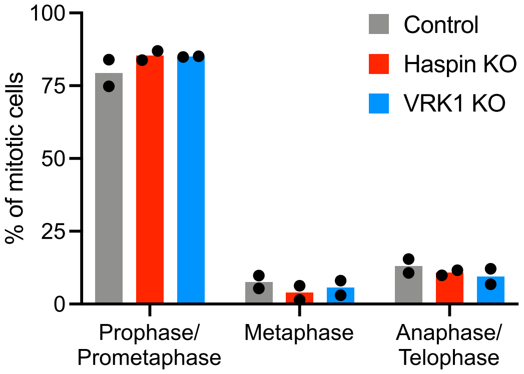

Supplementary Figure 3
Control, Haspin KO, and VRK1 siRNA-treated HeLa cells were fixed and stained with DAPI to visualize DNA, and with antibodies to H3T3ph and H3S10ph (n = 2 independent experiments). The stage of mitosis was determined by DAPI staining for all cells from prophase to early telophase.

A GSG2 (Haspin) gene (2 bp insertion in exon 1)

```
      100                           110                           120
      R  W  K  L  R  A  R  P  S  L  T  V  T  P  R  R  L  G  L  R  A  R  P  P  Q    K  C
WT  AGCGCTGGAAGCTGCGAGCTCGCCCAAGCCTAACCGTGACCCCAAGACGCCTGGGGCTGCGAGCTCGGCCCCCGC--AGAAGTGC
    |||||||||||||||||||||||||||||||||||||||||||||||||||||||||||||||||||||||||||  ||||||||
KO  AGCGCTGGAAGCTGCGAGCTCGCCCAAGCCTAACCGTGACCCCAAGACGCCTGGGGCTGCGAGCTCGGCCCCCGCAAAGAAGTGC
      R  W  K  L  R  A  R  P  S  L  T  V  T  P  R  R  L  G  L  R  A  R  P  P  Q  R  S  A

             130                           140                           150
    S  T  P  C  G  P  L  R  L  P  P  F  P  S  R  D  S  G  R  L  S  P  D  L  S  V  C  G  Q
WT  AGCACACCCTGCGGCCCGCTCCGACTTCCGCCCTTCCCCAGCCGCGACTCCGGCCGCCTCAGCCCGGACCTCAGCGTGTGCGGCC
    |||||||||||||||||||||||||||||||||||||||||||||||||||||||||||||||||||||||||||||||||||||
KO  AGCACACCCTGCGGCCCGCTCCGACTTCCGCCCTTCCCCAGCCGCGACTCCGGCCGCCTCAGCCCGGACCTCAGCGTGTGCGGCC
     A  H  P  A  A  R  S  D  F  R  P  S  P  A  A  T  P  A  A  S  A  R  T  S  A  C  A  A

                  160                           170                           180
      P  R  D  G  D  E  L  G  I  S  A  S  L  F  S  S  L  A  S  P  C  P  G  S  P  T  P  R
WT  AGCCCAGGGACGGCGACGAGCTGGGCATCAGTGCCTCCCTGTTCAGCTCTCTGGCCTCGCCCTGCCCCGGGTCCCCAACGCCAAG
    |||||||||||||||||||||||||||||||||||||||||||||||||||||||||||||||||||||||||||||||||||||
KO  AGCCCAGGGACGGCGACGAGCTGGGCATCAGTGCCTCCCTGTTCAGCTCTCTGGCCTCGCCCTGCCCCGGGTCCCCAACGCCAAG
    S  P  G  T  A  T  S  W  A  S  V  P  P  C  S  A  L  W  P  R  P  A  P  G  P  Q  R  Q  G

                       190                           200                           210
     D  S  V  I  S  I  G  T  S  A  C  L  V  A  A  S  A  V  P  S  D  L  H  L  P  E  V  S
WT  GGACAGTGTCATCTCGATCGGCACCTCCGCCTGTCTGGTTGCAGCCTCAGCCGTCCCGAGCGGCCTCCACCTCCCAGAAGTCTCC
    |||||||||||||||||||||||||||||||||||||||||||||||||||||||||||||||||||||||||||||||||||||
KO  GGACAGTGTCATCTCGATCGGCACCTCCGCCTGTCTGGTTGCAGCCTCAGCCGTCCCGAGCGGCCTCCACCTCCCAGAAGTCTCC
      T  V  S  S  R  S  A  P  P  P  V  W  L  Q  P  Q  P  S  R  A  A  S  T  S  Q  K  S  P

                            220                           230                           240
    L  D  R  A  S  L  P  C  S  Q  E  E  A  T  G  G  A  K  D  T  R  M  V  H  Q  T  R  A  S
WT  CTGGACCGAGCATCTCTCCCCTGCTCCCAGGAGGAAGCGACAGGAGGAGCCAAGGACACCAGGATGGTCCACCAAACCCGCGCCA
    |||||||||||||||||||||||||||||||||||||||||||||||||||||||||||||||||||||||||||||||||||||
KO  CTGGACCGAGCATCTCtcccctgctcccaggaggaagcgacaggaggagccaaggacaccaggatggtccaccaaacccgcgcca
     W  T  E  H  L  S  P  A  P  R  R  K  R  Q  E  E  P  R  T  P  G  W  S  T  K  P  A  P

                                 250
      L  R  S  V  L  F  G  L  M  N  S  G  T  P  E  D  S
WT  GCCTCAGGTCAGTTCTCTTTGGCCTTATGAACTCAGGAACCCCTGAGGATT
    |||||||||||||||||||||||||||||||||||||||||||||||||||
KO  gcctcaggtcagttctctttggccttatgaactcaggaacccctgaggatt
    A  S  G  Q  F  S  L  A  L  *
```

B VRK1 gene (11 bp deletion in exon 5)

```
                                       100                           110
                              Q  K  W  I  R  T  R  K  L  K  Y  L  G  V  P  K  Y  W  G  S
WT  ACAAAGATCTGTTTTAATTTGTAGTTCAGAAATGGATTCGTACCCGTAAGCTGAAGTACCTGGGTGTTCCTAAGTATTGGGGGTC
    ||||||||||||||||||||||||||||||||||||||||||||||||||||           ||||||||||||||||||||||
KO  ACAAAGATCTGTTTTAATTTGTAGTTCAGAAATGGATTCGTACCCGTAAGCT-----------GTGTTCCTAAGTATTGGGGGTC
                              Q  K  W  I  R  T  R  K  L             C  S  *

             120
    G  L  H  D  K  N  G  K  S
WT  TGGTCTACATGACAAAAATGGAAAAAGGTAAAAATATGT
    |||||||||||||||||||||||||||||||||||||||
KO  TGGTCTACATGACAAAAATGGAAAAAGGTAAAAATATGT
```

Supplementary Figure 4

DNA and predicted amino acid sequences of Haspin and VRK1 genes in wild type (WT C631) HAP1 cells (top lines) compared with **A**, Haspin KO (HZGHC000047c016) and **B**, VRK1 KO (HZGHC000073c014) HAP1 cells (bottom lines). Numbering refers to amino acid position. Grey shaded regions are exons. Red regions highlight deletions or insertions resulting in frame shifts. Sequencing data from Horizon Discovery.

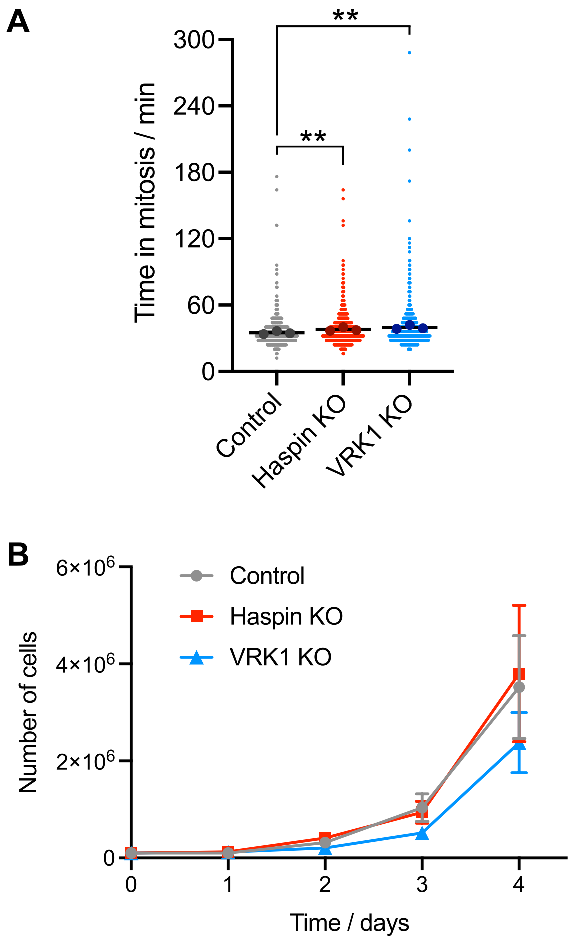

Supplementary Figure 5

A. The duration of mitosis (from nuclear envelope breakdown to anaphase onset) in control, Haspin KO and VRK1 KO HAP1 cells was determined by live imaging. Between 145 and 342 cells per condition were evaluated from three separate fields of view in each of three independent experiments. Small symbols represent individual cells, and large symbols show the mean duration of mitosis in each of the three experiments. Black bars show the means of these means (n = 3 independent experiments), ** adjusted p < 0.001 by one-way ANOVA followed by Dunnett's multiple comparisons test.

B. The proliferation of control, Haspin KO and VRK1 KO HAP1 cells was measured by cell counting. Error bars show means +/- SD (n = 3 independent experiments).

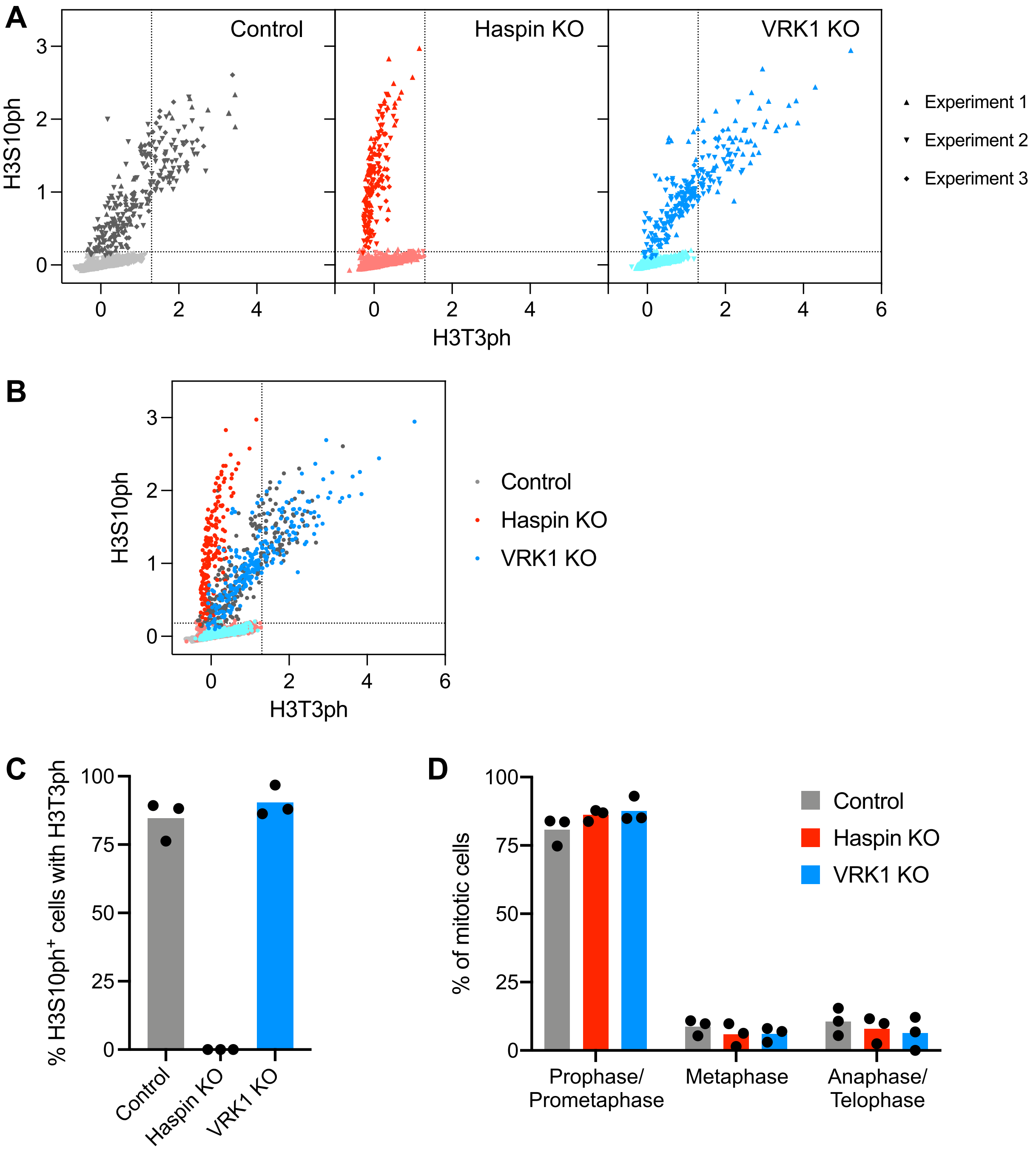

Supplementary Figure 6

A. Quantification of the intensity of H3T3ph and H3S10ph immunofluorescence in all cells within asynchronous control, Haspin KO and VRK1 KO HAP1 populations (as in Figure 5). Darker symbols indicate cells in mitosis as identified by automated outlier analysis of H3S10ph (see Methods); lighter symbols are cells in interphase. The results from 3 separate experiments are shown. An average of 1700 cells from each cell line was measured in each experiment (15269 total cells).

B. The same data as in (A), overlaid to allow comparison.

C. Control, Haspin KO and VRK1 KO HAP1 cells were fixed and stained with DAPI to visualize DNA, and with antibodies to H3T3ph and H3S10ph. The percentage of all cells with H3S10ph staining that had detectable H3T3ph was visually scored (n = 3 independent experiments).

D. The stage of mitosis was determined by DAPI staining for all cells from prophase to early telophase (n = 3 independent experiments).

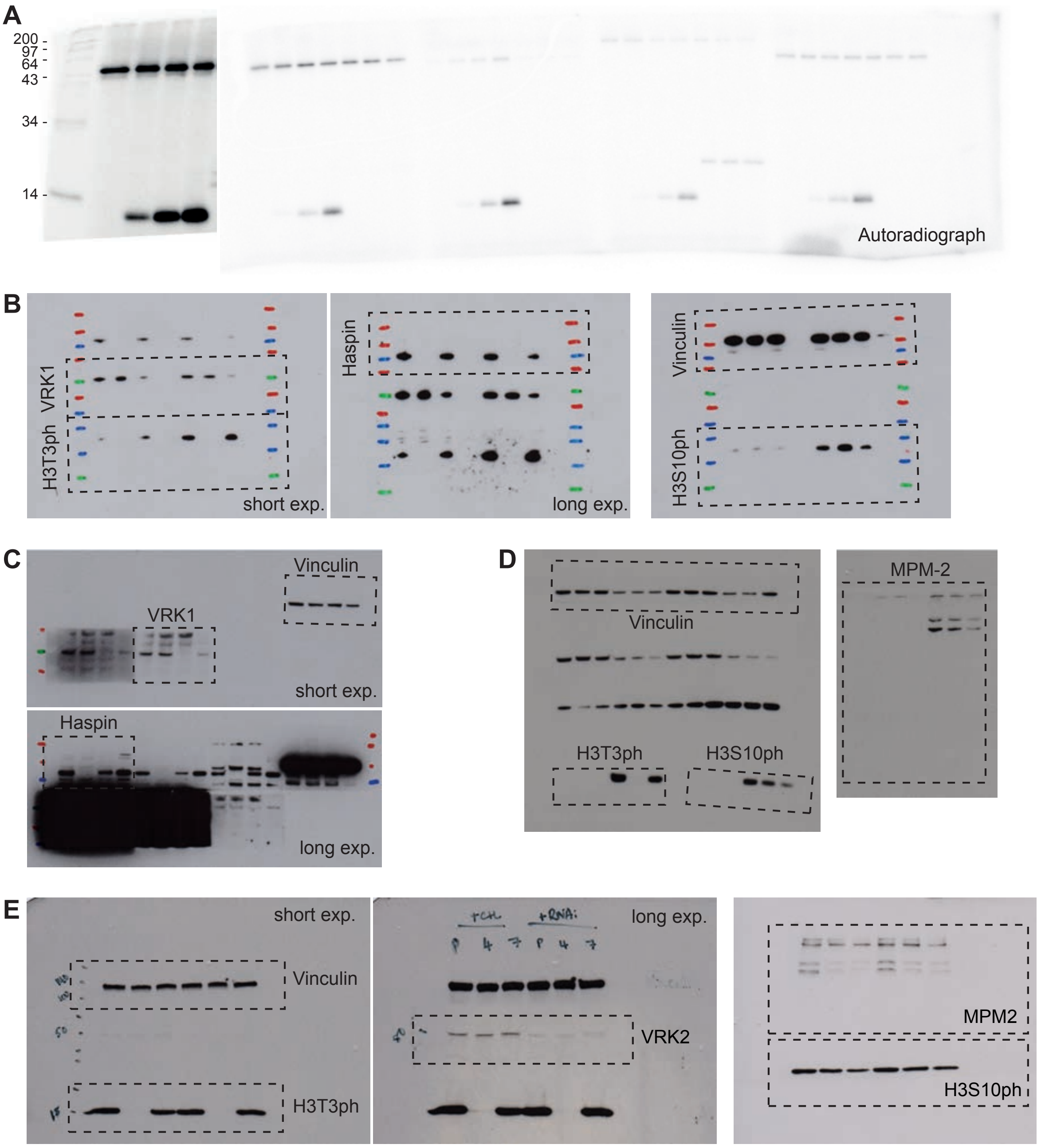

Supplementary Figure 7

Original gels and blots used in the main figures. Most blots were cut into multiple sections prior to probing. Dashed line boxes indicate the extent of the membrane portions immunoblotted with the antibodies indicated (blot background was too low to see membrane edges). Cropped images from within these regions are shown in the main figures.

A, for Figure 1C (note that a portion of an additional similar gel is shown on the left that contains ^{14}C-molecular weight markers); **B**, for Figure 3B; **C**, for Figure 4A; **D**, for Figure 4B; **E**, for Figure 6.

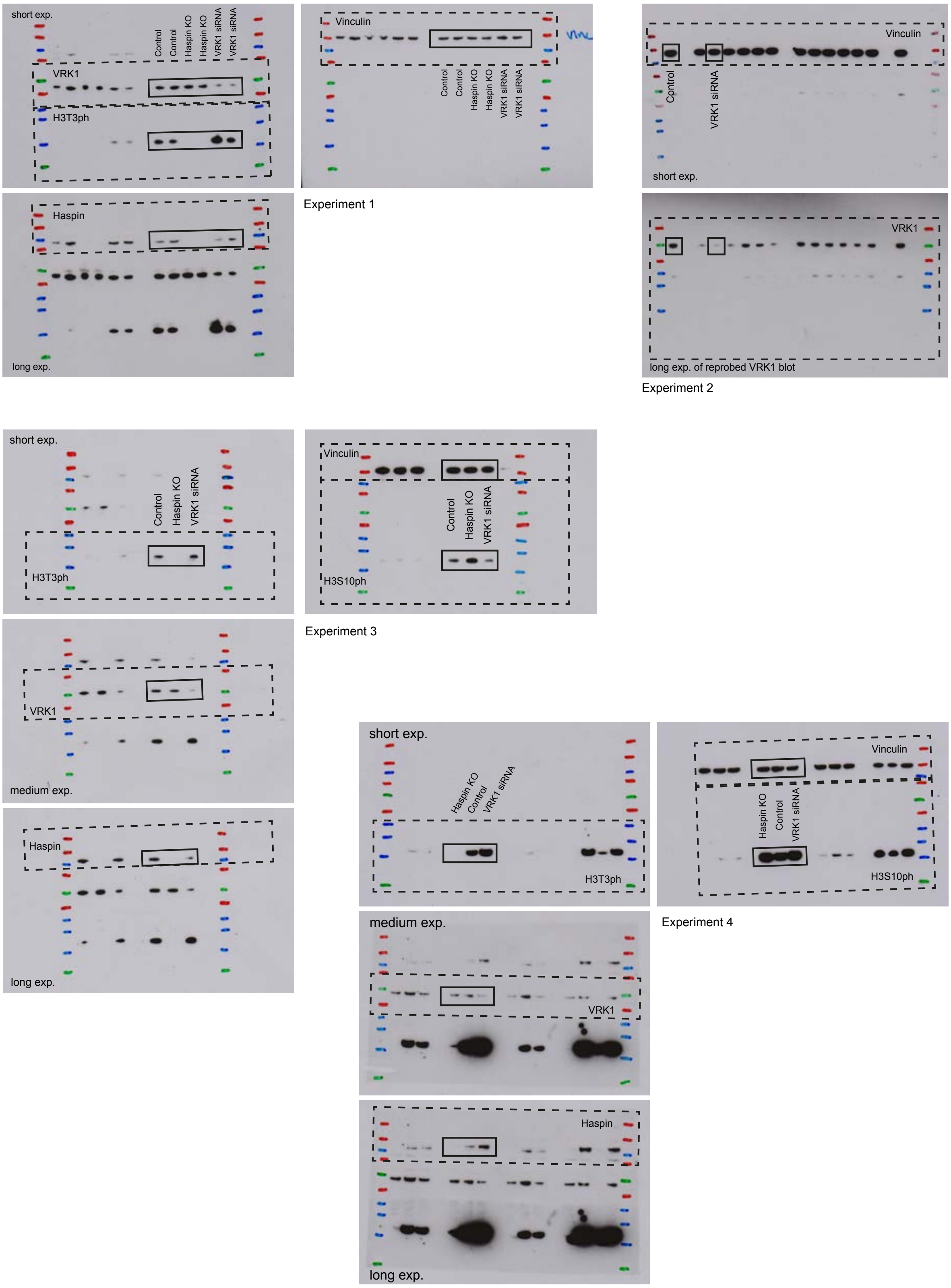

Supplementary Figure 8

Original blots for the quantifications shown in Supplementary Table 1A. Most blots were cut into multiple sections prior to probing. Dashed line boxes indicate the extent of the membrane portions immunoblotted with the antibodies indicated (blot background was too low to see membrane edges). Quantified bands within these regions are shown within solid line boxes.

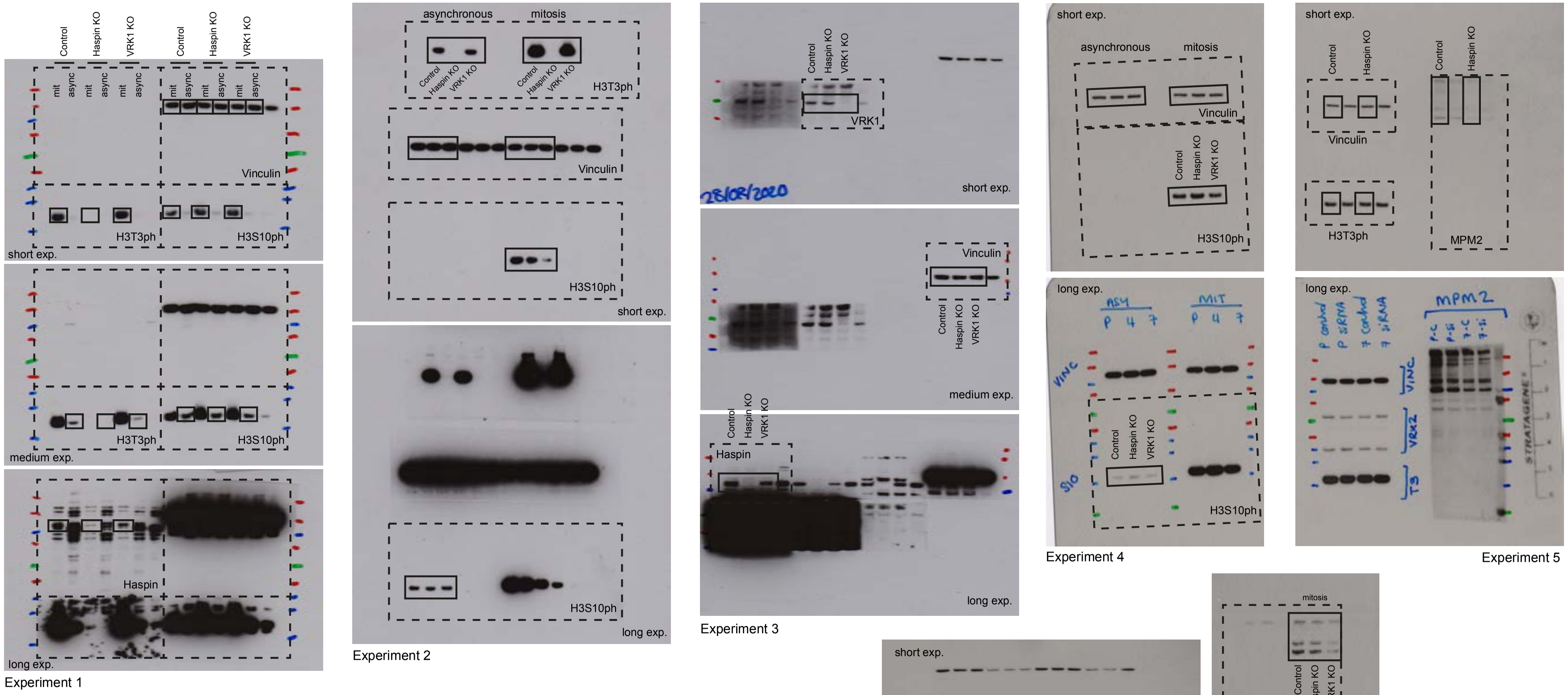

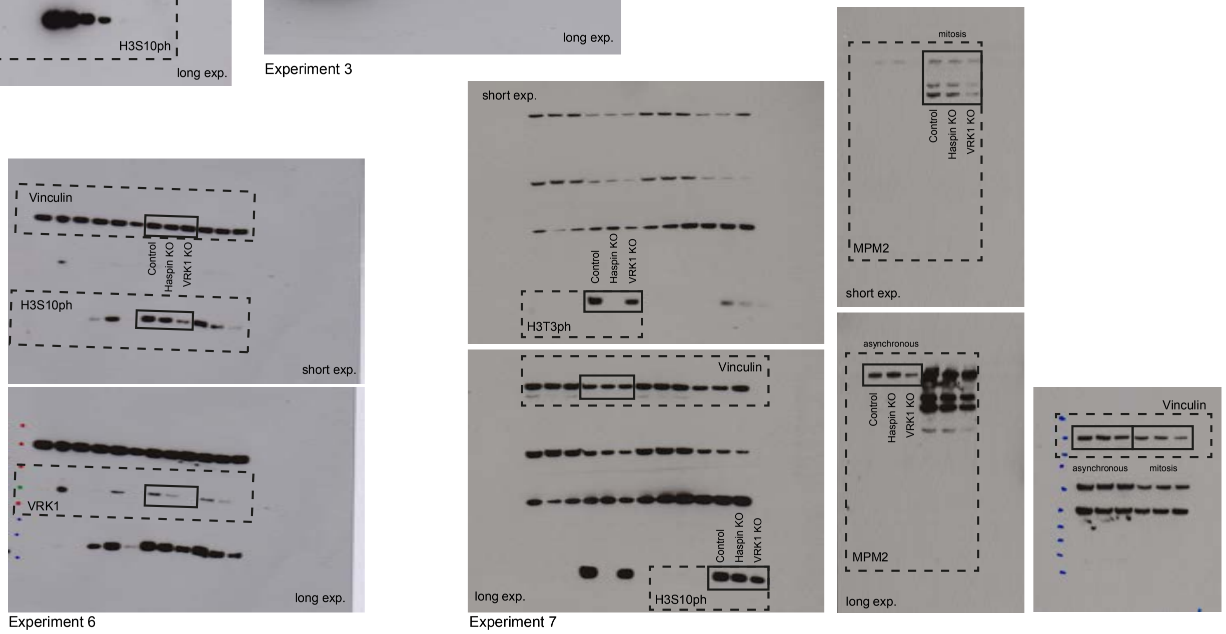

Supplementary Figure 9
Original blots for the quantifications shown in Supplementary Table 1B. Most blots were cut into multiple sections prior to probing. Dashed line boxes indicate the extent of the membrane portions immunoblotted with the antibodies indicated (blot background was too low to see membrane edges). Quantified bands within these regions are shown within solid line boxes.

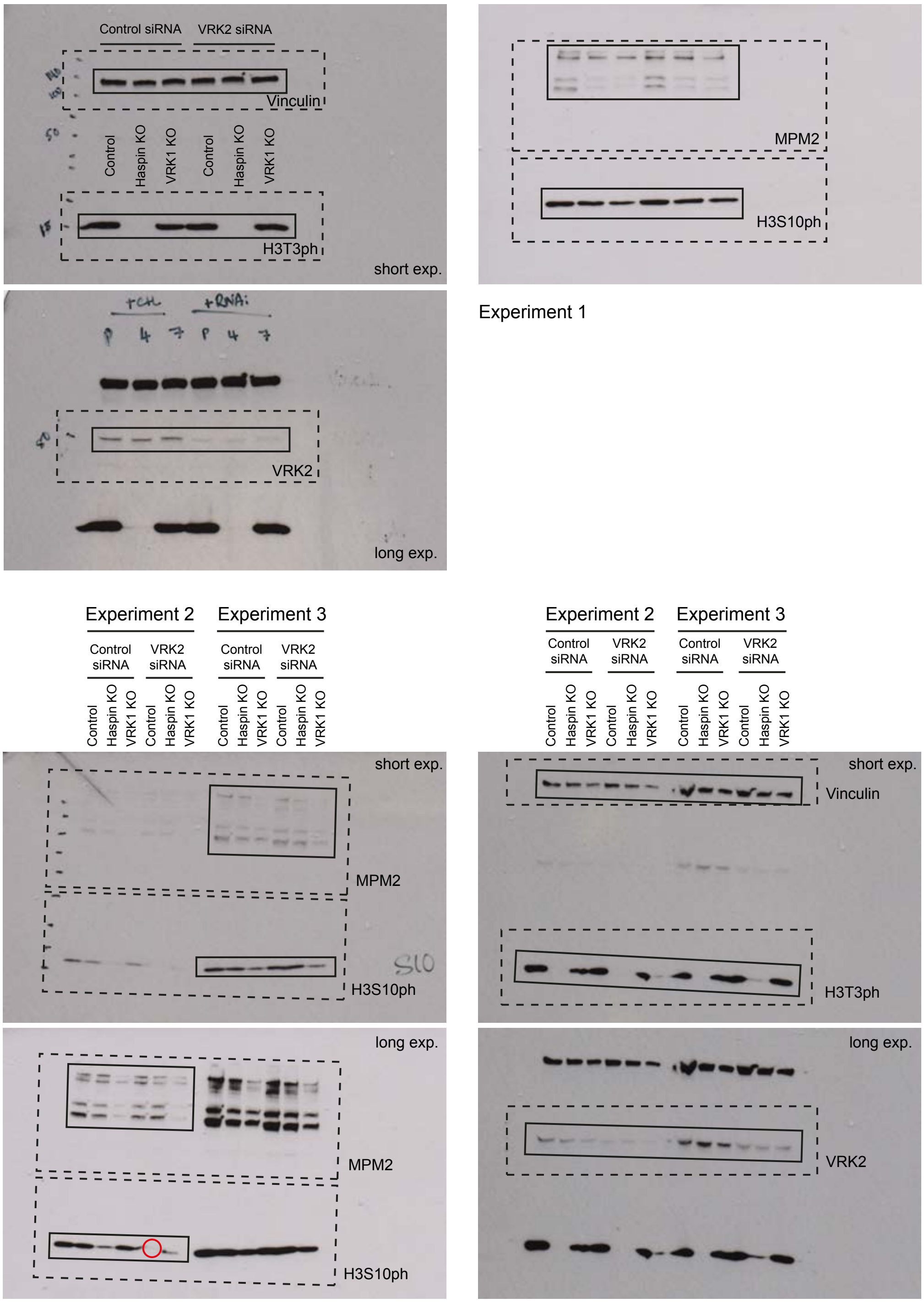

Supplementary Figure 10

Original blots for the quantifications shown in Supplementary Table 1C. Most blots were cut into multiple sections prior to probing. Dashed line boxes indicate the extent of the membrane portions immunoblotted with the antibodies indicated (blot background was too low to see membrane edges). Quantified bands within these regions are shown within solid line boxes. The red circle indicates an air bubble that prevented transfer. This lane of the H3S10ph blot was discarded.

A

	Control cell			Haspin KO			VRK1 RNAi		
	mean	SD	n	mean	SD	n	mean	SD	n
VRK1	100	na	5	99	14	5	23	10	5
Haspin	100	na	4	2	3	4	145	172	4
H3T3ph	100	na	4	2	0.5	4	168	32	4
H3S10ph	100	na	2	163	63	2	105	59	2

B

	Control cell			Haspin KO			VRK1 KO		
	mean	SD	n	mean	SD	n	mean	SD	n
VRK1	100	na	2	92	22	2	-4	4	2
Haspin	100	na	2	3	8	2	91	6	2
H3T3ph	100	na	3	1	2	6	98	34	6
H3S10ph	100	na	8	110	29	8	79	41	8
MPM-2	100	na	3	100	34	2	53	17	3

C

	Control cell/Control RNAi			Haspin KO/Control RNAi			VRK1 KO/Control RNAi			Control cell/VRK2 RNAi			Haspin KO/VRK2 RNAi			VRK1 KO/VRK2 RNAi		
	mean	SD	n	mean	SD	n	mean	SD	n	mean	SD	n	mean	SD	n	mean	SD	n
VRK2*	100	na	3	100	na	3	100	na	3	27	13	3	28	12	3	34	18	3
VRK2	100	na	3	119	59	3	105	48	3	27	13	3	31	11	3	32	18	3
H3T3ph	100	na	3	4	1	3	123	36	3	104	10	3	6	7	3	144	49	3
H3S10ph	100	na	3	109	15	3	72	8	3	101	6	3	114	25	2	71	7	3
MPM-2	100	na	3	79	21	3	44	8	3	103	17	3	98	30	3	41	7	3

Supplementary Table 1

Quantification of immunoblotting results.

A. Comparison of VRK1, Haspin, H3T3ph, and H3S10ph levels in control HeLa cells, Haspin KO HeLa cells, and VRK1 RNAi-treated HeLa cells.

B. Comparison of VRK1, Haspin, H3T3ph, H3S10ph, and MPM-2 levels in control, Haspin KO, and VRK1 KO HAP1 cells.

C. Comparison of VRK2, Haspin, H3T3ph, H3S10ph, and MPM-2 levels in control, Haspin KO, and VRK1 KO HAP1 cells with and without VRK2 RNAi treatment. In the row marked with an asterisk (*), VRK2 amounts were standardized separately for each of the three cell types.
