# Supplementary material for: Dissecting the roles of Haspin and VRK1 in histone H3 phosphorylation during mitosis
Source: Sci Rep. 2022 Jul 1;12:11210. doi: 10.1038/s41598-022-15339-8 (PMC9249732; doi:10.1038/s41598-022-15339-8)
Supplement: Supplementary file 5 — Supplementary Legends. [file 41598_2022_15339_MOESM5_ESM.pdf]

Supplementary Data 1. The custom library.
Details of the 140 kinase inhibitors constituting the custom library.

Supplementary Data 2. Kinases profiled.
The table lists all the kinases profiled in the Davis, Anastassiadis, Gao, and Federov datasets used in this study. The kinase names used are those in the original published profiling studies. In addition, the HGNC symbols for each kinase are provided. The total numbers of kinases profiled are shown at the bottom.

Supplementary Data 3. KiPIK screening results (related to Figure 2).
